# ALOPECIA AREATA IS NOT ASSOCIATED WITH *HELICOBACTER PYLORI*

**DOI:** 10.4103/0019-5154.48979

**Published:** 2009

**Authors:** Hisham Zayan Abdel Hafez, Ayman Mohamed Mahran, Eman M Hofny, Dalia Abdel Aziz Attallah, Doaa Sameer Sayed, Heba Rashed

**Affiliations:** *From the Department of Dermatology, Assiut University Hospital, Egypt*

**Keywords:** *Alopecia areata*, *etiology*, *Helicobacter pylori*

## Abstract

**Background:**

Alopecia areata (AA) is an immune-mediated form of hair loss that occurs in all ethnic groups, ages, and both sexes. *Helicobacter pylori* has been associated with many extra-digestive dermatological conditions. The causal relation between alopecia areata and *Helicobacter pylori* is discussed in this study.

**Materials and Methods:**

We have screened for the presence of *H. pylori* in patients with AA, in order to determine any potential role in its patho-physiology. We have prospectively studied 31 patients with alopecia areata and 24 healthy volunteers of similar gender, for the presence of *H. pylori* stool antigen (HpSAg).

**Results:**

Optical density values for *H. pylori* infection was positive in 18 of the 31 patients evaluated (58.1%), while in 13 patients, the values did not support *H. pylori* infection (41.9%). In the control group, 10 of the 24 (41.7%) had positive results. Within the group of alopecia areata, there was no significant difference between HpSAg positive and negative patients.

**Conclusions:**

The results have shown that a relation between *Helicobacter pylori* and alopecia areata is not supported. We advise that *H. pylori* detection need not to be included in the laboratory work up of alopecia areata.

## Introduction

Alopecia areata (AA) is an immune-mediated form of hair loss that occurs in all ethnic groups, ages, and both sexes; with an estimated lifetime risk of 1.7% among the general population.[1] Circumstantial evidence indicates that AA may be an autoimmune disease. The most direct evidence is provided by the histological finding that in early stages, the lower ends of hair follicles are surrounded by an infiltrate of lymphocytes.[2] The hypothesis of the autoimmune nature of AA is supported not only by the coexistence with other autoimmune diseases, but also by the presence of auto-antibodies against thyroid constituents, gastric parietal cells, and smooth muscle cells.[3,4] Furthermore, the identification of antibodies against normal anagen scalp hair follicles in the serum of patients with AA supports this hypothesis.[5]

Since *Helicobacter pylori* (*H. pylori*) identification in 1983,[6] an increasing amount of knowledge has accumulated, with this agent having been directly involved in the pathogenesis of several gastro-duodenal pathologies.[3] *H. pylori* is considered to be a noninvasive organism that is essentially confined to the gastric mucosa. In addition to their direct injury to target tissues, infectious agents might exert their deleterious effects indirectly by interfering with the immune system.[7] This bacterium has been associated with certain extra-digestive dermatological conditions, including chronic urticaria, rosacea,[8] Henoch-Schönlein purpura, Sweet syndrome, systemic sclerosis, and atopic dermatitis.[3]

On the basis of these studies and considering the fact that AA is a disease of unknown origin, we have screened for the presence of *H. pylori* in patients with AA, in order to determine any potential role in its patho-physiology.

## Materials and Methods

This study was designed to determine the incidence of *H. pylori* infection among AA patients and in healthy controls. From March 2006 through December 2006, at the Dermatology Outpatient Clinic, Assiut University Hospital, we prospectively evaluated patients with AA and healthy subjects (control group, with comparable age and sex) without clinical evidence of any skin disorder or any history of dermatological problems. Patients or healthy subjects who had ever received treatment for *H. pylori* infection were excluded from the study.

A clinical history and thorough physical examination were obtained for all the subjects. Following this, the subjects were invited to participate in the study, which was conducted according to the Declaration of Helsinki principles and was approved by the local Ethics Committee. Those who accepted to participate in the study were referred to perform a stool antigen test for *H. pylori* (HpSAg).

A total of 31 patients with AA were enrolled in the study (21 male and 10 female). Demographic (age, sex, duration) and gastro-intestinal symptoms were recorded using a structural questionnaire. Twenty four healthy volunteers of similar gender and age distribution were selected as the control group.

*H. pylori* antigens were detected using a kit supplied by Premier Platinum HpSA, manufactured by Meridian Diagnostics Inc. USA (Cat no 601348-045), based on enzyme immunoassay for *in vitro* qualitative detection of *H. pylori* antigens in human stool.[9] The samples were collected on the same day of diagnosis and were stored and frozen at -20°C. According to the manufacturer's instructions, the cut off optical density (OD) values used were as follows: <0.140 negative, 0.140-0.159 equivocal and >0.160 is positive. Equivocal results were considered negative.

### Statistical analysis

Statistical analysis was performed using the SPSS 10.0 statistical software package, with continuous variables being presented as mean, standard deviation and range, and discrete variables being presented as percentage and 95% confidence interval (95% CI). Statistical tests employed in the analysis were unpaired student's t-test and the chi-square test.

## Results

Thirty one patients with AA were compared with 24 healthy volunteers of the control group. At the time of the initial visit, 48.4% were presented with AA at the front of the scalp, 45.2% with AA at the back of scalp, 9.7% at the beard, 12.9% at the temporal area, 6.5% at the eye brows and 3.2% at the moustache. The percentage of patients who presented with a single lesion of AA was 77.4%, while 22.6% presented with multiple lesions. The size of the lesions ranged from 0.2 to 11.8 cm^2^. The duration of the disease ranged from two days to one year, with 71% having a progressive course and 29% having a stationary one. The clinical characteristics of alopecia areata are summarized in Table 1.

**Table 1:** Clinical characteristics of Alopecia areata

	Descriptive statistics
Duration	Mean + SD: 2.35 + 2.90 (months)
	Range: 2 days - 1
Course	Progressive: 22 (71.0%)
	Stationary: 9 (29.0%)
Site	Front of scalp: 15 (48.4%)
	Back of scalp: 14 (45.2%)
	Beard: 3 (9.7%)
	Temporal: 4 (12.9%)
	Eye brows: 2 (6.5%)
	Moustache: 1 (3.2%)
Size	Mean + SD: 4.74 + 2.77 (cm^2^)
	Range: 0.8-11.8
Sites multiplicity	Single: 24 (77.4%)
	Multiple: 7 (22.6 %)

**Figure 1:**
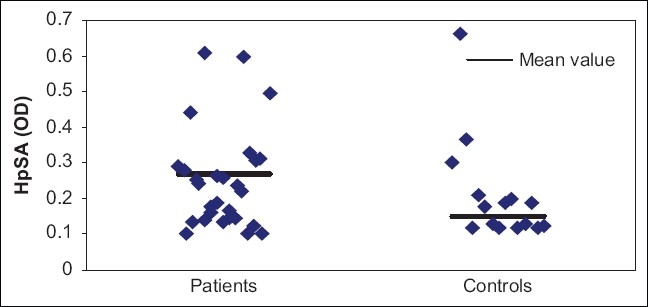
Scaterrogram for HpSA among both groups of the study

Both the groups were comparable in terms of age (mean 24.48 vs 27.50 years) and sex (67.7% vs 58.3% males in both). Optical density values for *H. pylori* infection was positive in 18 of all the 31 patients evaluated (58.1%), while in 13 patients optical density values did not support *H. pylori* infection (41.9%). In the control group, 10 of 24 (41.7%) had positive results [Figure 1]. Although the mean of *H. pylori* stool antigen (HpSAg) was higher in the AA group, it did not reach statistical significance between the two groups.

Within the group of alopecia areata, there was no significant difference noticed with regard to age, gender, duration, course, size and multiplicity between HpSAg positive and negative patients, as shown in Table 2.

**Table 2:** Characteristics of alopecia areata patients in relation to the presence of *H. pylori*

Characteristics	Presence of *Helicobacter pylori* (n = 18)(%)	Absence of *Helicobacter pylori* (n = 13)(%)	*P*
Age (years): Mean ± SD	25.28 ± 12.17	23.38 ± 7.81	NS
Sex incidence: No. (%)			NS
Males	12 (66.7)	9 (69.2)	
Females	6 (33.3)	4 (30.8)	
Duration (months):			NS
Mean ± SD	2.44 ± 2.74	2.23 ± 3.23	
Course:			NS
Progressive	12 (66.7)	10 (76.9)	
Stationary	6 (33.3)	3 (23.1)	
Size (cm^2^): Mean ± SD	4.89 ± 2.76	4.53 ± 2.89 NS	NS
Sites multiplicity:			NS
Single	13 (72.2)	11 (84.6)	
Multiple	5 (27.8)	2 (15.4)	

NS: Not significant

Figures in parenthesis are in percentage.

## Discussion

Circumstantial evidence indicates that AA may be an autoimmune disease. The familial predisposition for developing autoimmune diseases and the association with auto-antibodies in patients with AA is analogous to findings in other autoimmune diseases and strengthens the possibility that AA is an autoimmune disease.[2] It is not clear, however, whether specific anti-follicle auto-reactivity occurs, although antibodies have not been found by direct immunofluorescence in the affected scalp.[10]

Since *H. pylori* identification in 1983, a putative pathogenetic role has been ascribed to this bacterium in several extra digestive diseases, including rosacea, ischemic heart disease, and diabetes mellitus.[11] In addition to their direct injury to target tissues, infectious agents might exert their deleterious effects indirectly by interfering with the immune system.[8]

De Luis *et al*. have published two articles showing that the seroprevalence of *H. pylori* is significantly higher in patients with insulin-dependent diabetes mellitus[4] and autoimmune atrophic thyroiditis.[8] They express the hypothesis that *H. pylori* antigens might be involved in the development of these two autoimmune diseases or that autoimmune function in these diseases may increase the likelihood of an *H. pylori* infection. On the basis of these studies and considering the fact that AA is a disease of unknown origin, we tried to determine whether there might be an association of *H. pylori* infection with AA.

Although *H. pylori* infection is essentially confined to the gastric mucosa, a single study has searched for an association between this bacterium and alopecia areata. This trial has shown no significant difference in the seroprevalence of *H. pylori* infection between patients with AA and healthy controls.[12]

In our study, given the high reliability of stool testing for the verification of *H. pylori*, we have found no evidence for an increased association between *H. pylori* infection and alopecia areata, supporting the previous study by Rigopouos *et al*.[12] and declaring that *H. pylori* cannot be incriminated in the pathogenesis of alopecia areata.

## Conclusions

Our results proposed no causal link between *H. pylori* infection and alopecia areata. Indeed, it would be difficult to imagine how a chronic bacterial infection of the gastric mucosa might initiate or perpetuate a chronic inflammatory skin disorder. Based on these, we advise that *H. pylori* detection need not to be included in the laboratory work up of alopecia areata.
